# Haemosporidian parasite prevalence, parasitemia, and diversity in three resident bird species at a shrubland dominated landscape of the Mexican highland plateau

**DOI:** 10.1186/s13071-016-1569-3

**Published:** 2016-05-27

**Authors:** María Teresa Reinoso-Pérez, Julio César Canales-Delgadillo, Leonardo Chapa-Vargas, Lina Riego-Ruiz

**Affiliations:** Instituto Potosino de Investigación Científica y Tecnológica A.C., Camino a la Presa San José #2055, Colonia Lomas 4. Sección, San Luis Potosí, S.L.P. C.P. 78216 México; CONACYT-Instituto de Ciencias del Mar y Limnología Estación El Carmen, Universidad Nacional Autónoma de México, Playa Norte y López Mateos s/n, Ciudad del Carmen, C.P. 24121 Campeche México

**Keywords:** Haemosporidians, Birds, Arid zones, Habitat degradation

## Abstract

**Background:**

Studies of avian haemosporidians allow understanding how these parasites affect wild bird populations, and if their presence is related to factors such as habitat loss, degradation and fragmentation, and climate change. Considering the importance of the highland Plateau of Mexico as part of the North American bird migratory route and as a region containing important habitat for numerous bird species, the purpose of this study was to document haemosporidian species richness and how habitat degradation, bird body condition, and distance from water sources correlate with bird parasitemia.

**Methods:**

We assessed the presence of avian haemosporidians in three resident bird species through microscopy and PCR amplification of a fragment of the haemosporidian cytochrome *b* gene. Average parasitemia was estimated in each species, and its relationship with habitat degradation through grazing, bird body condition and distance from water bodies was assessed.

**Results:**

High levels of parasitemia were recorded in two of the three bird species included in this study. Four lineages of haemosporidians were identified in the study area with nearly 50 % prevalence. Areas with highly degraded shrublands and villages showed higher parasitemia relative to areas with moderately degraded shrublands. No strong relationship between parasitemia and distance from water bodies was observed. There were no significant differences in prevalence and parasitemia between the two bird species infected with the parasites. Two of the sequences obtained from the fragments of the parasite’s cytochrome *b* gene represent a lineage that had not been previously reported.

**Conclusions:**

Haemosporidian diversity in arid zones of the Mexican highland plateau is high. Shrubland habitat degradation associated to the establishment of small villages, as well as tree extraction and overgrazing in the surroundings of these villages, significantly enhances parasitemia of birds by haemosporidians.

**Electronic supplementary material:**

The online version of this article (doi:10.1186/s13071-016-1569-3) contains supplementary material, which is available to authorized users.

## Background

Haemosporidan parasites are transmitted by 17 genera of blood-sucking insects of the order Diptera [1, 2]. The study of these parasites allows the understanding of various aspects of avian ecology. Parasites indirectly reduce host fitness by decreasing their reproductive success and increasing breeding energy cost [3]. Therefore, parasitism by haemosporidians constitutes a selective force on bird populations. Consequently, interactions between birds and haemosporidian parasites have become a model of host-parasite relationships in ecology, conservation and vertebrate management [2, 4].

Around the world, presence of haemosporidians has been evaluated in tropical and temperate regions. Worldwide and within regions, prevalence values are highly variable, from 0 to 100 % depending on species, but in general, prevalence values tend to be slightly higher, around 50 to 80 %, in tropical regions [5–8], in comparison with temperate regions of North America and Europe where typical prevalence is around 50 and 70 %, respectively [9–12]. Diversity of haemosporidian lineages is also high throughout the globe but tend to be higher in continents than in islands, and in tropical than in temperate regions [13]. However, only a few haemosporidian parasite studies have focused on the diversity of these organisms in dry regions [3, 4, 14]. Knowledge related to factors influencing patterns of bird parasitism by haemosporidians in dry regions of the Americas is rather limited. One study in Arizona reported prevalence values from 0 to 80 %, and parasitemia by *Haemoproteus* related to nesting height, but no infections by *Plasmodium* and *Leucocytozoon*, and speculated that these results could be related to a number of factors including resistance to parasites, ecological barriers, or the absence of *Plasmodium* and *Leucocytozoon* vectors in this environment due to the limited water availability [4]. Furthermore, a study in arid zones of Venezuela reported 41 % haemosporidian prevalence and high species diversity including seven lineages from the genus *Plasmodium* and ten from *Haemoproteus*. It was also reported that humidity and temperature influence vector distribution and abundance [3].

There is also evidence that human-driven habitat changes and the increase in global temperatures are facilitating the expansion of parasite distribution ranges. Infection prevalence and parasite species richness respond to interactions between biotic and abiotic factors [3, 8, 15]. These processes create ecological niches that facilitate the establishment of vector populations [15]. Results of comparisons between anthropogenic and relatively undisturbed habitat types have suggested that habitat type and quality may influence the distribution and behavior of the vectors of these parasites, consequently affecting haemosporidian parasite dispersion patterns [15–17]. Others have reported that parasite dispersal may be influenced by habitat features such as proximity to water and climate change [18].

The establishment of villages, as well as tree extraction and overgrazing in the surroundings of these villages are common practices that have induced shrubland habitat degradation in a large proportion of the Mexican high plateau [19]. Therefore, we aimed at evaluating the effect of habitat degradation on parasitemia in resident bird species, and hypothesized that increasing levels of habitat degradation would be correlated with increasing parasitemia [19]. To further understand the underlying mechanisms associated with the effects of habitat degradation on parasitemia through potential negative effects on bird immune system, we assessed the potential correlation between individual bird body condition and parasitemia. Finally, because the construction of artificial ponds for the capture and storage of rainwater is a recurring practice in our study area and in most of the Mexican high plateau, we also evaluated the effect of proximity to water ponds on parasitemia. Our study reports the first records of haemosporidian parasites in avian populations from the highland plateau of Mexico and the effects of environmental variables on parasitemia. Thus, our results may function as the basis for future studies investigating host-parasite interactions between birds and haemosporidians in the region.

## Methods

### Study area

**Fig. 1:**
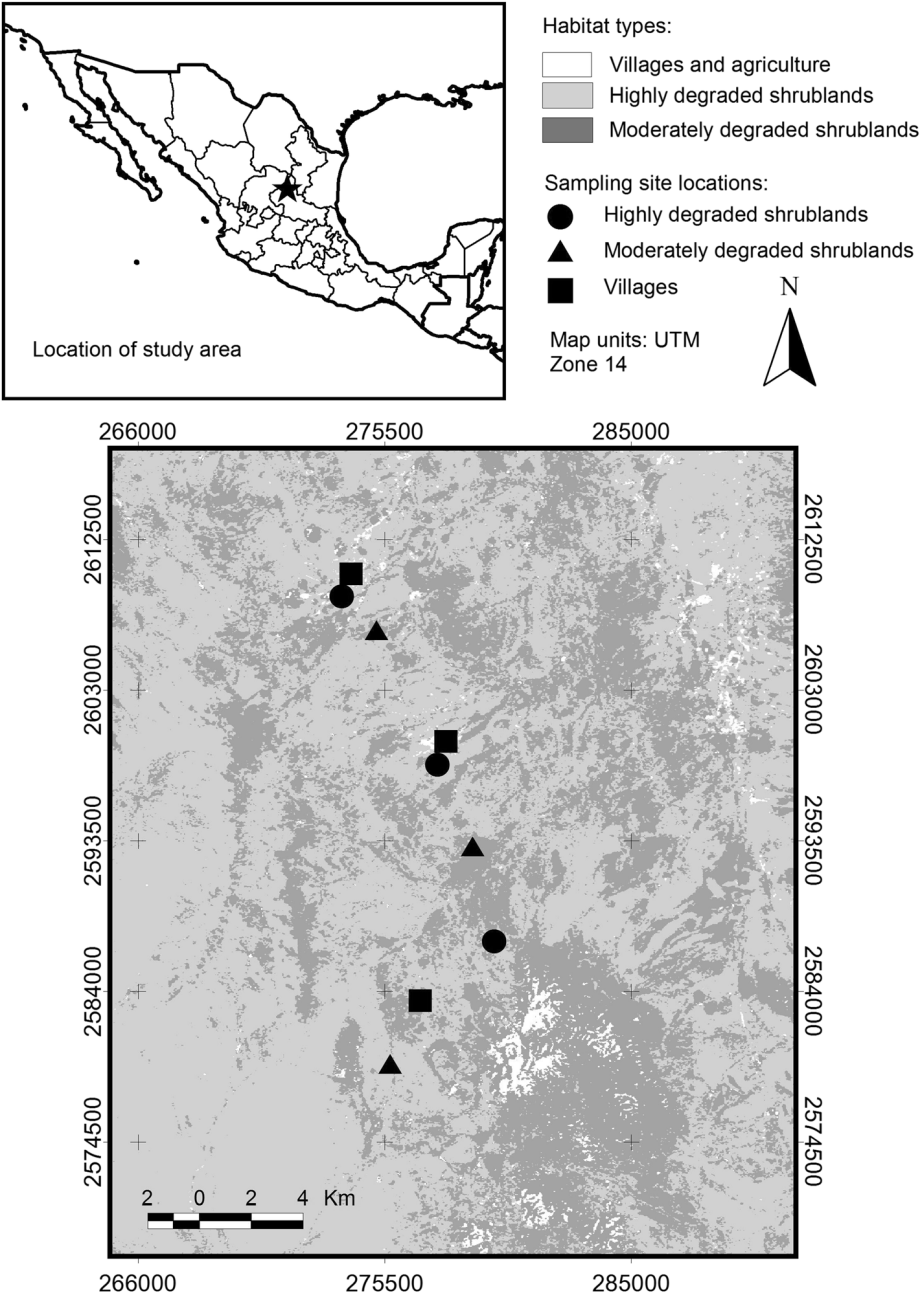
Study area. Location of sampling sites at the “Ixtlera” region in northern San Luis Potosi, Mexico

The study area is located between the municipalities of Charcas and Catorce, in the Mexican state of San Luis Potosi. Three communal-owned areas (ejidos) were included in the study: La Cardoncita (LC), Presa de Santa Gertrudis (PSG), and Guadalupe Victoria (GV) (UTM: E271793.37 N2610708.64, E278059.47 N2599525.12, E276057.75 N2582923.98 Zone 14 N). All study sites were embedded in a region known as “Zona Ixtlera” (Fig. 1), where trees of the genus *Yucca* have been historically extracted to obtain fiber, locally known as “Ixtle”, which is used for manufacture of ropes and other crafts. The vegetation in the area is characterized by the presence of rosette microphylous scrublands dominated by *Yucca* spp. in the tree layer, and *Larrea tridentata* and *Flourensia cernua* in the shrub layer [20]. A high diversity of species from the family Cactaceae is also present. The area contains gentle slopes and low mountains that facilitate storm water runoff to the lowlands. Annual rainfall averages 350 mm, and annual average temperature ranges from 10 to 20 °C [21, 22]. Altitude ranges from 2000 to 2300 m.

In the early 1930’s, a large portion of the expropriated land within the Mexican territory was given to local communities, and thus tenure in these lands was established in the “ejido” scheme. At this time, regional economies incentivized *Yucca* extraction for “Ixtle” production. Overgrazing was also established as an additional form of local economic subsistence. Because these economic activities continue to date, shrubland habitat degradation through the growth of villages, extraction of tree species, and overgrazing in their surrounding still prevail, thus creating a habitat gradient consisting of at least the following habitat types [19]: (i) “Isotal”, thereafter moderately degraded shrublands, where grazing and tree extraction is moderate; (ii) microphylous shrublands dominated by creosote bush in which the tree layer has been eliminated and intense goat grazing is ongoing, thereafter highly degraded shrublands; and (iii) villages where the natural vegetation has been almost completely removed and substituted by houses and unpaved roads. Three sampling sites representative of these levels of habitat degradation were established in each ejido. In each study site, bird communities were surveyed, and blood samples from birds were taken according to the methods described below.

### Sampling

In the non-breeding (August to November) season of 2012, and during the reproductive (March to May) and non-breeding (September to November) seasons of 2013, birds were captured in each of the nine sampling sites. For this purpose, twenty 2.5 × 12 m ornithological mist nets were placed at random locations in each sampling site with the following restrictions: nets should be located at no less than 300 m from all habitat edges in order to avoid possible edge effects, all nets could be revised by one person in a period of 15 to 20 min, and no two nets would be located facing each other. This allowed us to revise nets quickly in order to avoid injuries to birds, and to maintain independence among nets. From each individual of *Haemorhous mexicanus* (house finch), *Melozone fusca* (canyon towhee), and *Campylorhynchus brunneicapillus* (cactus wren) a blood sample of approximately 200 μl was taken by jugular vein puncture [23]. Blood samples were stored in eppendorf tubes with 1 ml of Longmire buffer (100 mM Tris–HCl pH 8.0, 100 mM EDTA, 10 mM NaCl, 0.5 % SDS) which allows storage of blood samples at room temperature without compromising DNA integrity [24]. Additionally, wing cord and body mass of all captured individuals were measured following the procedures recommended by Ralph et al. [25].

### Microscopy and blood smears

Blood smears were prepared following standard techniques for haemosporidian studies [1]. Slides were examined using an optical Motic BA400 microscope with a 100× objective amplification. Parasitemia was quantified for each smear based on 100 random fields, approximately 150 erythrocytes/field = 15,000 total erythrocytes, in which the number of infected cells was recorded. Parasite identification procedures followed the characteristics described by Valkiūnas [1], specifically examining the state of development of micro and macro-gametocytes, meronts, and the presence of pigment granules.

### DNA extraction

DNA was extracted from each blood sample using the DNeasy blood & tissue isolation kit (Qiagen, Venlo Netherlands) according to the manufacturer’s instructions. To verify DNA integrity, extractions were screened on 1 % agarose gels and the quality and quantity of the extracted DNA was assessed with a NanoDrop 2000 Spectrophotometer (Thermo Fisher Scientific, Inc.).

### Molecular diagnosis by PCR

Presence or absence of haemosporidian parasites was determined in each sample through amplification of fragments of approximately 480 bp of the parasite cytochrome-*b* gene using HaemF (5'-ATG GTG CTT TCG ATA TAT GCA TG-3') and HaemR2 (5'-GCA TTA TCT GGA TGT GAT AAT GGT-3') primers [26]. 25 μl PCR reactions were carried out; each reaction contained 20–50 ng of total DNA, 1× PCR buffer II (100 mM Tris–HCl, pH8.3, 500 mM KCl), 1.5 mM MgCl_2_, 0.125 mM each dNTP, 0.6 μM of each primer and 0.6 U of AmpliTaq (Applied Biosystems, Foster City, California). The reactions were conducted according to the protocol developed by Bensch et al*.* [26] as follows: initial denaturation at 94 °C for 3 min, 30 cycles of denaturation at 94 °C for 30 s, annealing at 50 °C for 30 s and extension at 72 °C for 45 s, and final extension step of 72 °C for 10 min. All reactions were carried out in a 2720 (ABI, Foster, CA) or MJMini (BioRad, Hercules, California thermocycler) thermal cyclers. PCR products were examined by 1 % agarose gel electrophoresis and the ones with the expected size were purified for sequencing. Haemosporidian parasite prevalence was estimated using the R package “Prevalence” [27] that uses a sensitivity, the probability that a truly infected individual reacts positively, and specificity, the probability that an individual truly uninfected yields negative results, diagnostic test. This test corrects the bias due to possible false positives and false negatives [28], obtaining an unbiased estimate of the real prevalence of infection through Bayesian inference using the following equation:

$$ \uppi = \mathrm{P}\left(\mathrm{D} = 1\right) = \left(\mathrm{T}\mathrm{P} + \mathrm{F}\mathrm{N}\right)/\left(\mathrm{T}\mathrm{P} + \mathrm{F}\mathrm{P} + \mathrm{F}\mathrm{N} + \mathrm{T}\mathrm{N}\right) $$

where: π is the true prevalence, P is the apparent prevalence, D is the status of infection, TP is true positive, FN is false negative, FP is false positive, and TN is true negative.

### Sequence analysis

Sequencing was performed on a 3130 Genetic Analyzer sequencer (Applied Biosystems, Foster City, California) in the National Laboratory of Agricultural, Medical, and Environmental Biotechnology (LANBAMA-IPICYT). The sequences obtained were compared with sequences deposited in public databases such as NCBI and MalAvi [29] to identify the species or genera of organisms causing infection in the samples. All sequences were deposited in the GenBank database (Accession numbers KR818942-KR819003) (for details see Additional file 1). Following the criteria published by Durrant et al*.* [9], sequences that diverged by two or more nucleotides were considered as different lineages, and to corroborate these as new lineages, we submitted our sequences to the MalAvi database. (GenBank accession numbers KR819002 and KR819003 for CatoC00400_MELFUS01 and CatoC00443_MELFUS01, respectively). Similarity between the obtained and the previously known sequences were determined through a Neighbor Joining (NJ) analysis. Evolutionary distances between sequences were calculated using the three-parameter model of Tamura, specifying the distribution of rate variation among sites γ = 0.263. To construct the cladogram, 1000 replicates were used in a bootstrap test to calculate the percentage of association between taxa. All these analyses were conducted in MEGA version 6 [30]. Because in our sequence analysis we could not detect co-infections, we complemented this analysis with microscopy techniques.

### Hydrological analysis

Through visual interpretation of satellite imagery from Google Earth of the study area, all permanent and temporary water bodies present in the study area were located and geo-referenced. Water bodies in our study region are characterized by showing a smooth texture and being bright in aerial imagery. Using QGIS 2.0.1 Dufour software [31], these water bodies were digitized to calculate the total area covered by water. We also used the Dufour QGIS 2.0.1 software [31] and Google Earth imagery to measure distances between each sampling site and the nearest water body.

### Statistical analysis

Differences in prevalence among bird species were assessed with a Chi-square test. Parasitemia was compared between bird species using a *t* test for independent samples specifying the negative binomial distribution for these count data. These tests were only applied to the two species yielding positive parasitemia.

For each bird species, we fitted a series of Generalized Linear Models (GLM’s) to assess the effects of factors influencing parasitemia including habitat degradation [19], distance from nearest water body, and bird body condition. Body condition was determined for each individual bird according to the Peig and Green escalated mass ratio [32], which is based on the relationship between body weight and a linear measure of length (e.g. wing length) of each individual. This has recently proved to be a more reliable indicator of true organismal condition than the most popular index based on the residuals of the ordinary least squares regression of body mass *vs* any measure of body size. The number of parasitized cells was used as the dependent variable. Because evidence of over dispersion was found, the negative binomial distribution was used for these count data.

We used Akaike’s Information Criterion corrected for small sample sizes (AIC_c_), and Akaike weights (*w*_*i*_) to evaluate support for several *a priori* generalized linear models of our hypotheses related to effects influencing parasitemia [33]. Our set of *a priori* models explaining parasitemia for the house finch included: 1) a null model with no explanatory variables, 2, 3, and 4) models of habitat degradation, distance from water bodies, and body condition index as the only explanatory variables, 5) a multiplicative model including habitat degradation, bird body condition, and the interaction of these two variables, and 6) a multiplicative model including distance from nearest water body, bird body condition, and the interaction of these two variables. For the canyon towhee we did not consider a model containing bird body condition as the only explanatory variable because the algorithm failed to converge, presumably due to the relatively small sample size. We did not include a multiplicative model containing habitat degradation and distance from water bodies for any of the studied species because results of an exploratory analysis indicated that there was autocorrelation between these two explanatory variables (one way ANOVA, *P* < 0.01), presumably because distance from water bodies tends to increase with increasing distance from villages. Therefore, distance from water bodies is smallest at villages, intermediate at highly degraded shrublands, and highest at moderately degraded shrublands. It is recommended that whenever two explanatory variables are autocorrelated, one of these variables should be dropped from the model [34]. The null model was used to determine if a random model with no explanatory variables generated a better model than inclusion of any of the remaining variables. We used AICc, Akaike differences (ΔAICc), and *w*_*i*_ [33] to rank models from most to least supported by the data. Then, to account for model-selection uncertainty, we calculated model-averaged weighted parameter estimates and their associated standard errors using *w*_*i*_ as weights as suggested by Burnham & Anderson [33]. Because some variables were more represented in our set of candidate models than others, we rescaled the *w*_*i*_ values following recommendations of Burnham & Anderson [33] to avoid possible model redundancy. Finally, we used weighted parameter estimates and weighted standard errors to construct graphs depicting the response in parasitemia to the variables that were present in the best-supported model and those receiving equivalent support from the data as the best-supported model (ΔAICc ≤ 2). All generalized linear models and chi square tests were conducted using R and the MASS library [35]. Because *w*_*i*_ values were rescaled, we obtained log likelihood values from R, and used these values to calculate AIC tables and average weighted parameter estimates in a spreadsheet adhering to the formulas published in Burnham & Anderson [33].

## Results

### Diagnostic tests

**Fig. 2:**
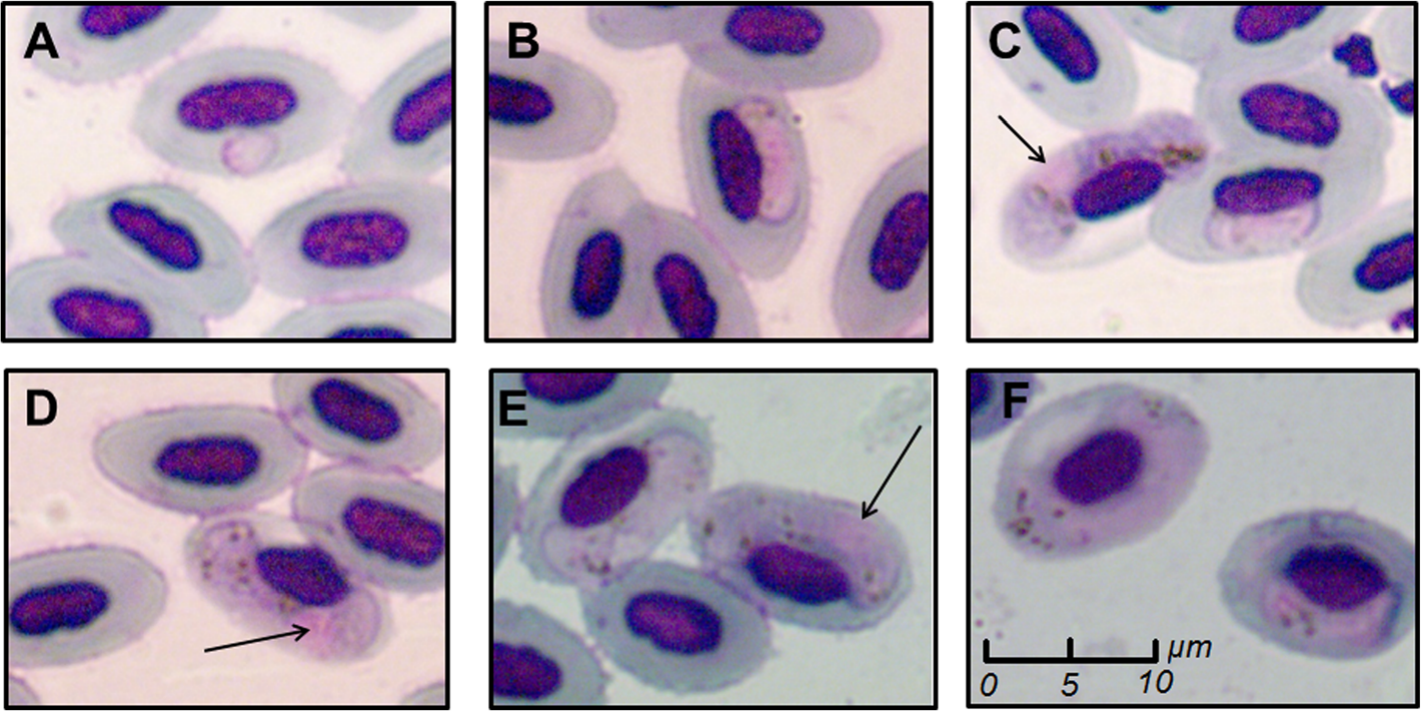
Infected erythrocytes. Blood smears showing erythrocytes infected by gametocytes of *Haemoproteus*. Early (**a**, **b**, **c**), and late (**c**, **d**, **e**, **f**) stages of development are shown. Nuclei of macrogametocytes are shown with arrows, whereas parasites without arrows are microgametocytes

Microscopy analysis revealed the presence of haemosporidian parasites in samples of two bird species, house finches and canyon towhees, whereas in cactus wren samples, only normal erythrocytes were observed. As is typical of the life-cycle of *Haemoproteus* spp., the asexual stages of these parasites, microgametocytes and macrogametocytes, within erythrocytes from infected birds were observed in most of our samples (Fig. 2). On the other hand, no meronts of *Plasmodium* were detected in red blood cells, but the presence of *Plasmodium* was evidenced by PCR amplification in two birds. Haemosporidian prevalence was 47.5 % for the canyon towhee, and 44.3 % for the house finch (Table 1).

**Table 1:** Diagnosis and estimation of haemosporidian parasite true prevalence in three bird species

Species	*n*	Positives		Prevalence (%)	$\overline{x}$ parasites/100 fields
		PCR	M		
Cactus wren	16	0	0	na	0
Canyon towhee	40	17	19	47.5	22.8 (3.6)
House finch	106	43	46	44.3	70.3 (12.3)

Number of bird blood samples from the “Ixtlera” region of northern San Luis Potosi yielding positive parasitism by haemosporidians. Estimates were obtained through PCR and observations of 100 fields (100×) through microscopy (M). *n* = total number of samples analysed by species. Prevalence = % of all samples from species yielding positive parasitemia. Average number of parasites found in 100 fields (100×) is reported (standard errors are presented in parenthesis)

**Fig. 3:**
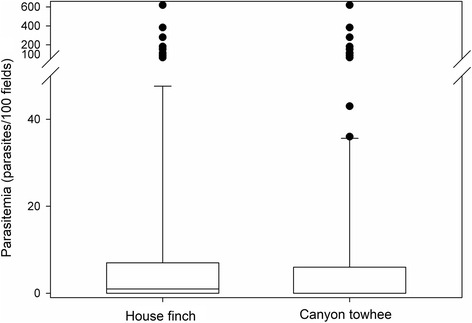
Parasitemia (number of parasites/100 fields). Parasites were recorded in blood smears of the house finch and the canyon towhee from the “Ixtlera” region in San Luis Potosi, Mexico. Black dots correspond to individuals yielding > 100 cells infected by haemosporidian parasites. *t* = 1.915, *df* = 55.408, *P* = 0.0606

In general, molecular diagnosis results matched those obtained from microscopy. However, the PCR amplification yielded six false negatives, four of which might be related to low concentration of parasite DNA that was corroborated through microscopy which yielded < 16 parasitized erythrocytes. Two other false negatives yielding somewhat less moderate parasitemia, consisting of 161 and 280 infected erythrocytes, respectively, were found and might be related to contamination or presence of inhibitors in the PCR reactions in spite of the fact that analyses were conducted using three replicates. Additionally, PCR amplification and sequencing of the DNA fragments of the parasites did not show cases of co-infection, but these were detected with microscopy examination in one of the house finch samples which revealed two cells infected with *Plasmodium* and eleven with *Haemoproteus*. The estimated prevalence of infection did not differ between the two species with positive cases (*χ*^2^ = 0.001, *df* = 1, *P* = 0.974). The house finch had the highest parasitemia, but this value did not differ significantly between the two species (*t* = -1.375, *df* = 141, *P* = 0.171, Fig. 3).

### Sequence analysis

**Fig. 4:**
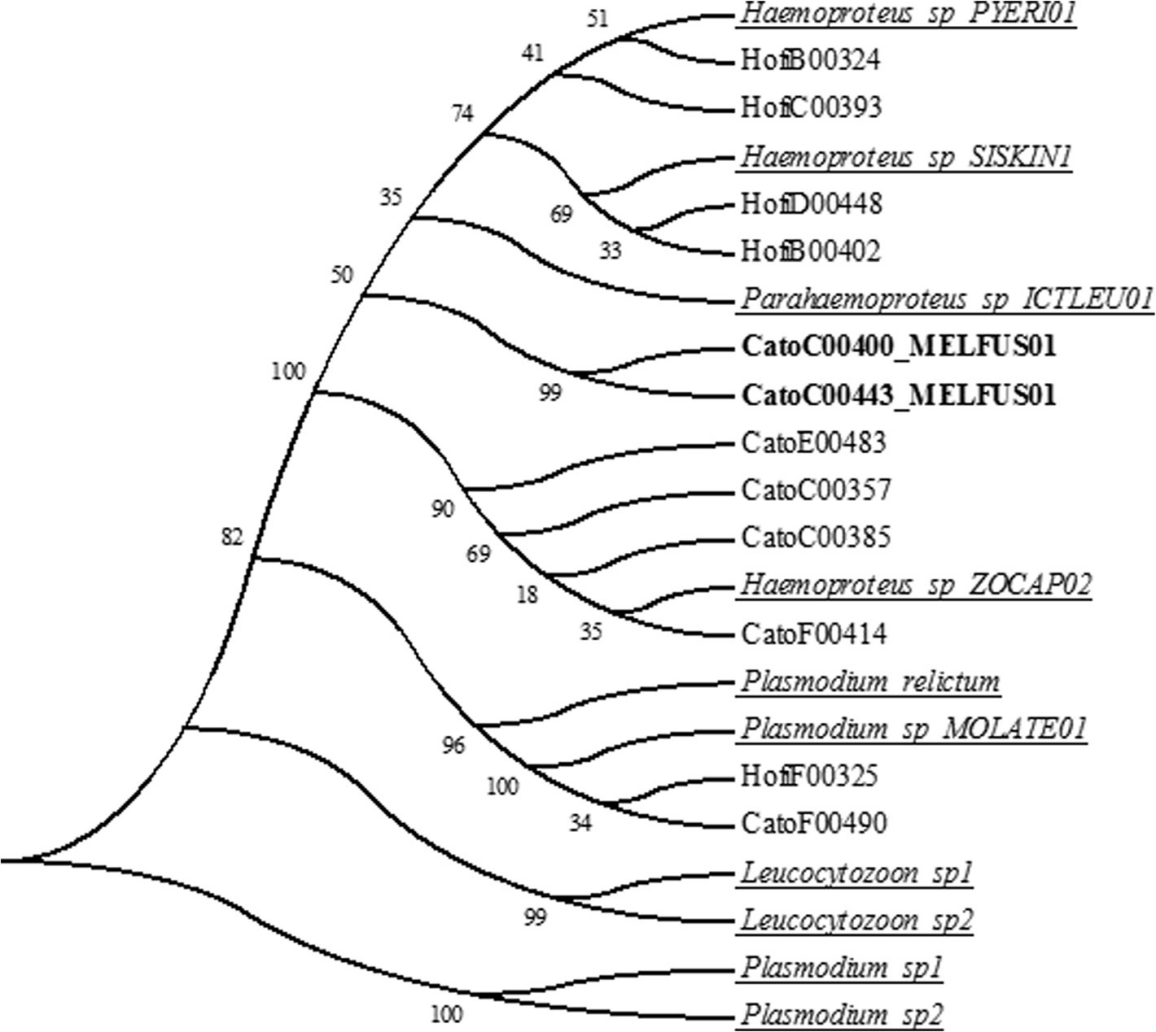
Specific and intraspecific diversity of haemosporidian parasites detected at the “Ixtlera” region. NJ cladogram showing specific and intraspecific diversity of haemosporidian parasite sequences obtained from samples yielding positive results from the house finch and canyon towhee from the “Ixtlera” region of San Luis Potosi. The sequences show similarity to the genus *Plasmodium* and subgenera *Haemoproteus* and *Parahaemoproteus*. CatoC00400 and CatoC00443 (in bold) correspond to a new lineage (MELFUS01). Only representative sequences are shown, for a full list of samples see Additional file 1. Bootstrap values are shown next to nodes. Underlined names correspond to reference sequences published on Genbank (see Additional file 1)

The amplicon sequencing revealed that in the study area house finches and canyon towhees get infected by at least three different lineages of the genus *Haemoproteus*. One lineage of the genus *Plasmodium* was detected in only one sample of each bird species, whilst two sequences from canyon towhee samples differ in eight nucleotides from the reference sequences. According to the method for delimiting lineages published by Bensch et al*.* [26, 29] these sequences represent a lineage that has not been previously reported. This was indeed corroborated as a new lineage by MalAvi (mbio-serv2.mbioekol.lu.se/Malavi/). The new lineage was designated as MELFUS01. Therefore the sequences belonging to this lineage are in a separate clade based on the similarity between sequences (Fig. 4).

### Effect of environmental variables

Through the hydrological analysis, we identified a total of 54 ponds with an average area of 1.2 ± 1.5 ha. Surface covered by water when all ponds are full is approximately 55 ha. Mean distance from water bodies was 1.51 km (standard error, SE = 0.13) for moderately degraded shrublands, 0.51 km (SE = 0.12) for highly degraded shrublands, and 0.24 km (SE = 0.04) for villages.

**Fig. 5:**
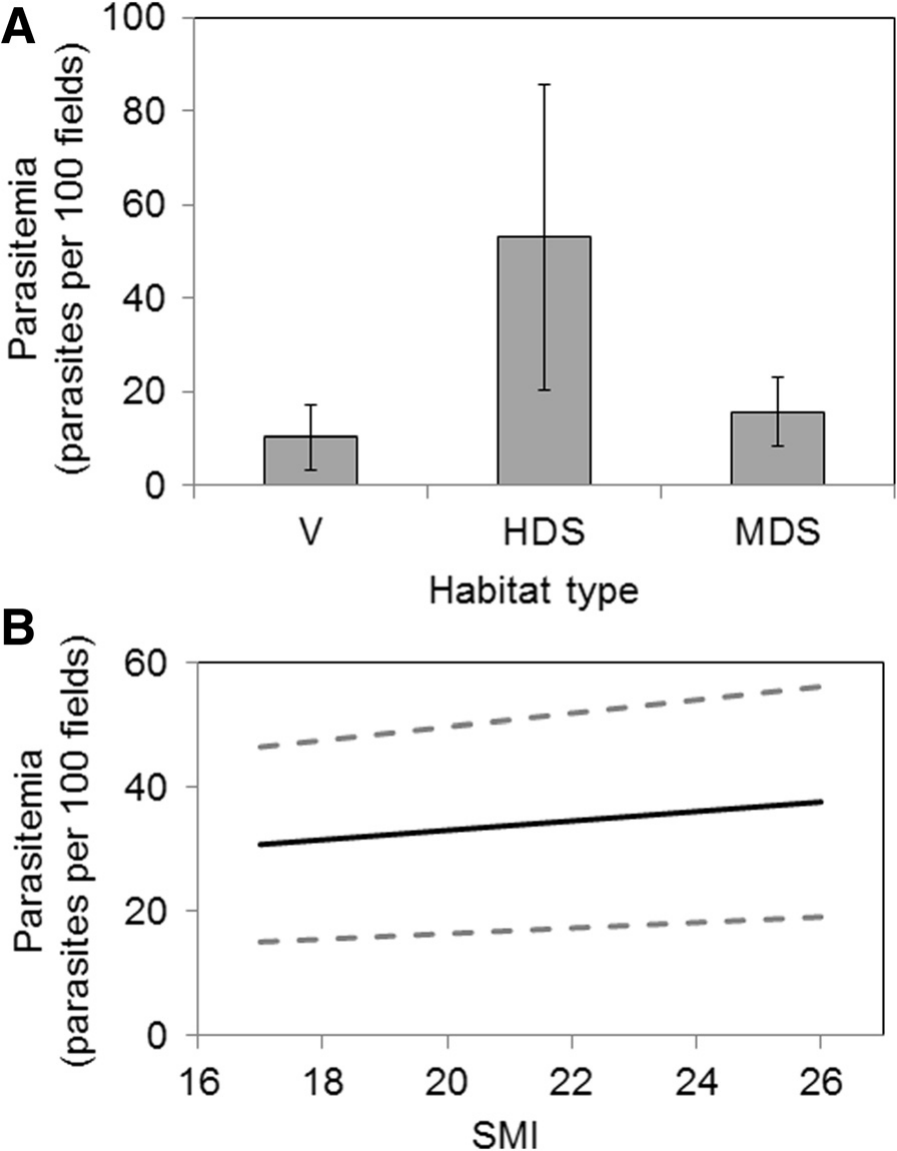
Effects of different factors on parasitemia in house finches. Effect of (**a**) habitat quality and (**b**) body condition on parasitemia (number of parasites/100 fields) in house finches from shrubland sites at the “Ixtlera” region of northern San Luis Potosi, Mexico. Error bars and dotted lines indicate ± one standard error around the mean

Though the null model was the one receiving the best support from the data for the house finch, the model containing multiplicative effects of body condition and habitat quality, and the model containing body condition index as the only explanatory variable received equivalent support from the data as the best supported model (ΔAICc < 2, Table 2). Based on these models, and on model-averaged parameter estimates (Table 3), parasitemia was highest in highly degraded shrublands, and lower in villages and in moderately degraded shrublands (Fig. 5a). In addition, parasitemia increased with increasing body condition, but the rate of this increase was almost negligible and standard errors were large (Fig. 5b).

**Table 2:** Model selection results for house finches

Model description	K	AICc	ΔAICc	wi
Null	2	572.4442049	0	0.629529619
H_q + SMI + H_q*SMI	7	573.5991571	1.154952288	0.117787773
SMI	3	574.0855941	1.641389263	0.092357414
Proximity	3	574.5604941	2.116289263	0.109254617
H_q	4	576.7143396	4.27013475	0.037216728
proximity + SMI + proximity*SMI	5	577.8798	5.435595146	0.013853848

Comparison of six a priori models of parasitemia in house finches from shrubland sites of different habitat quality at the “Ixtlera” region of northern of San Luis Potosi, Mexico. *Abbreviations*: *k* number of parameters, *AICc* Akaike information criterion adjusted for small sample sizes, *ΔAICc* Akaike differences, *wi* Akaike weights. *H_q* Habitat quality, *SMI* body condition index, proximity = distance from nearest water source. Null = a null model with only an intercept, but no explanatory variables

* Indicates interaction term

**Table 3:** Model-averaged weighted parameter estimates for house finches

Parameter	Level	Estimate	SE
Intercept		-0.51918097	1.75976561
Proximity		-0.75469164	1.02552681
SMI		0.76056617	0.31433299
H_q	Moderate	19.5947535	8.13021872
	High	39.7355876	11.284891
Interactions	SMI*Moderate	-1.171	0.4875
	SMI*High	-2.445	0.6794
	Proximity*SMI	0.3138	0.2804

Parameter estimates and standard errors for effects influencing house finch parasitemia. Parameter abbreviations as in Table 2. *SE* standard error

* Indicates interaction term

**Fig. 6:**
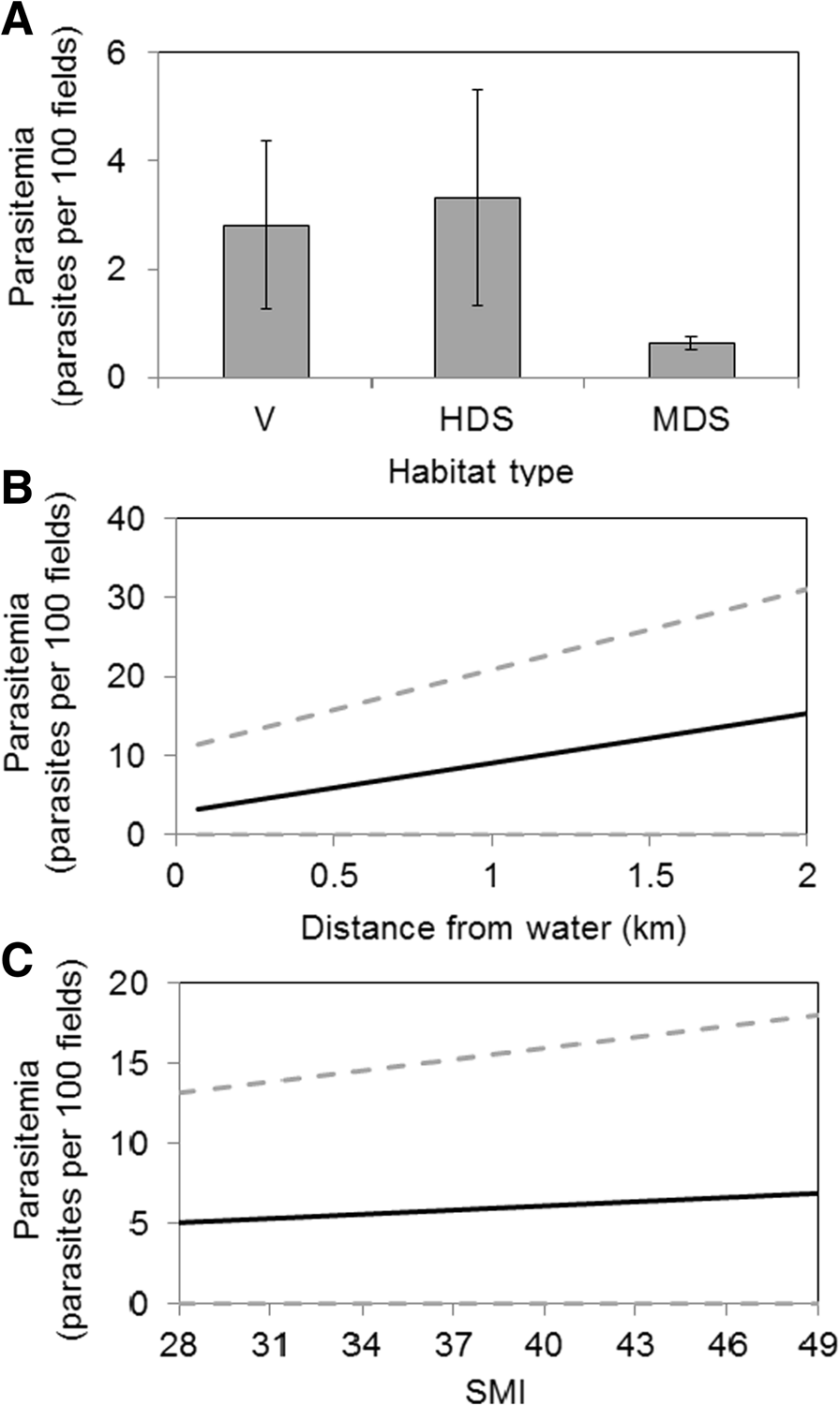
Effects of different factors on parasitemia in canyon towhees. Effect of (**a**) habitat quality, (**b**) distance from water bodies, and (**c**) body condition on parasitemia (number of parasites/100 fields) in canyon towhees from shrubland sites at the “Ixtlera” region of northern San Luis Potosi, Mexico. Error bars and dotted lines indicate ± one standard error around the mean

For the canyon towhee, the model containing habitat quality as the only explanatory variable was the one receiving the best support from the data. Three additional models receiving equivalent support from the data (ΔAICc < 2, Table 4) included the model containing proximity to ponds, the model containing multiplicative effects of habitat quality and bird body condition, and the model containing multiplicative effects of proximity to water bodies and body condition index. Based on the best supported models, and on model-averaged parameter estimates (Table 5), parasitemia for this species was highest in villages and highly degraded shrublands, and low at moderately degraded shrublands (Fig. 6a). Parasitemia increased with distance from water (although standard errors were quite large, Fig. 6b), and with increasing body condition index. This last increase, however, was very slight and standard errors were quite large (Fig. 6c).

**Table 4:** Model selection results for canyon towhees

Model description	K	AICc	ΔAICc	wi
H_q	4	186.88804	0	0.29269872
Proximity	3	187.564094	0.67605451	0.20874565
H_q + SMI + H_q*SMI	7	187.634857	0.74681754	0.20148905
proximity + SMI + proximity*SMI	5	187.7993	0.9112604	0.18558511
Null	2	190.204905	3.31686525	0.11148147

Comparison of six a priori models of parasitemia in canyon towhees from shrubland sites of different habitat quality at the “Ixtlera” region of northern of San Luis Potosi, Mexico. Abbreviations as in Table 2

* Indicates interaction term

**Table 5:** Model-averaged weighted parameter estimates for canyon towhees

Parameter	Level	Estimate	SE
Intercept		0.63890475	2.44356009
Proximity		3.5613453	3.05258657
SMI		0.0514524	0.13166858
H_q	Moderate	-3.34752357	2.82018321
	High	-7.81744062	3.64700034
Interactions	SMI*Moderate	0.3548	0.1412
	SMI*Highly	0.6107	0.18445
	Proximity*SMI	-0.2556	0.1418

Parameter estimates and standard errors for effects influencing canyon towhee parasitemia. Parameter abbreviations as in Table 2. *SE* standard error

* Indicates interaction term

## Discussion

The haemosporidian parasite prevalence that we recorded in two resident bird species of the highland plateau of San Luis Potosi are comparable to those previously reported in arid zones [3, 4, 14]. On the other hand, the prevalence values encountered are lower in comparison to those reported by other studies from tropical [5–8] and temperate [10–12] regions. Within individual sites, prevalence varies greatly among bird species. Therefore, future studies considering more bird species from dry areas will provide additional knowledge about within-region variation in prevalence values. In comparison to our results, Deviche et al*.* [4] failed at registering the genus *Plasmodium* in bird blood samples from arid Arizona habitats. At present it is difficult to understand the reasons for this difference; though water availability, which is sometimes necessary for the establishment of vector populations, may be a factor into play. Other factors such as exposure to parasites brought by migrants, resistance to parasites, ecological barriers, and other idiosyncrasies of both parasites and vectors may be important, and it is currently impossible to obtain more specific conclusions without further investigations simultaneously considering host and vector distribution and ecology within our study region. Notwithstanding, some literature indicates that *Plasmodium* prevalence is in general substantially smaller than that of *Haemoproteus* [36, 37], which is consistent with the lowest prevalence that we found for this genus and with the reports of Deviche et al. [4].

Haemosporidian infection prevalence is variable among bird species and even absent in some [1]. Lack of infection in the cactus wren may be related to one or many extrinsic and intrinsic factors. Potential extrinsic factors include absence of the specific host-parasite assemblage [38], which owes to evolutionary causes, and potential competitive exclusion of vectors by ectoparasites [39]. As reported for other species [4], behavior and some adaptations may be among intrinsic factors influencing our observations. Although the cactus wren tolerates some disturbance, it tends to settle far from human settlements, where vectors may be absent [38]. In addition, it is not highly dependent on sites with available water [40] as it seeks microhabitats with moderate temperatures and its activity decreases to prevent heat stress as the temperature reaches critical levels. Moreover, its physiological adaptations allow it to get water from food to maintain water balance even in extreme temperature conditions [41]. In addition, although cactus wren nesting is not well studied, it may be that it has nest defense strategies as the one reported for *Parus caeruleus* which uses herb species as a repellent against *Culex* mosquitoes to protect their nest [42]. Immune capacity may also benefit some species [43] and this could be the case of the cactus wren. Microbicidal capacity, for instance, which may be associated to nutrition quality and age [44, 45], could be a factor into play in our study as all captured cactus wren individuals were adults with no weight, size, or length below normal values for the species. In addition, as has been reported by Girard et al. [46] for other species, previous infection by other pathogens may have stimulated antibody production, which in turn may improve immune response to other infections. Although these could be important factors in a possible resistance of this species to infection by haemosporidians, these hypotheses should be experimentally tested. Such studies should include physiological tests in this species in order to understand its adaptation against such parasites, if any.

Because bird migration has contributed significantly to promoting the establishment of widespread haemosporidian parasite distributions throughout most of the planet [1], future studies should investigate haemosporidian parasites in blood samples from migratory birds using the area, and possible transmission between migratory and resident species, perhaps by banding birds, and simultaneously analyzing samples from residents, migrants, and the vector community. In such study, nestlings and juveniles from resident species that had fledged in our study sites during the times in which migrants were absent, could provide very valuable information. If migrants participate significantly in the parasite transmission process, prevalence in these juveniles would be expected to be significantly lower during the time in which migrants are absent. But caution must be taken in such a study because once a lineage is introduced by migrants, resident birds may function as reservoirs.

This study recorded a statistical relation between habitat quality and parasitemia of infected birds. This result might be related to different factors associated to habitat quality such as food availability. Damage to the immune system of birds inhabiting moderately degraded shrublands may also play a role in parasitemia. It has been previously documented that the same acute stress response that allows organisms to survive short-term environmental stressors, such as unusual weather events or the presence of a predator, via the release of corticosterone in birds, may lead to suppression of the immune system and the consequent increase in susceptibility to disease when the source of stress is prolonged, such as under anthropogenic disturbances [47]. Therefore, the higher parasitemia documented here at highly degraded shrublands could be the result of depressed immune system in these habitats. The correlation found between body condition and parasitemia provides additional evidence for the hypothesis that habitat degradation may influence the immune system, which in turn may promote higher parasitemia in house finches and canyon towhees inhabiting degraded habitats. This type of mechanism has been previously reported for other vertebrates [15, 16, 48]. Future studies investigating factors such as status of the immune system, food availability, vector densities in relation to different habitat features, as well as manipulative experiments could help disentangle these factors. Moreover, our results should be viewed with caution because parasitemia may be more related to the stage of infection in which each individual bird is recorded than to environmental factors.

The richness of parasite lineages found in this study is similar to that reported in other arid areas [3, 4]. The presence of different species depends on their environmental needs. Species of the genus *Leucocytozoon,* for instance, require the existence of well-oxygenated water flows for the successful development of the larval phase of at least some of their vectors such as *Similillium* spp. which are blood-sucking flies of the family Simulidae [49]. This is probably why this genus is uncommon in arid environments. According to our molecular diagnosis, four different lineages of the genera *Plasmodium* and *Haemoproteus* are present in our study area. These lineages have wide distributions; *Haemoproteus* sp. PYERY01 has been reported at many locations in the entire planet, except for Asia and the Antarctica [29, 50]. On the other hand, *Parahaemoproteus* sp. ICTLEU01 has been registered only at the American continent, specifically in North America, and at the Galapagos [51, 52]. Similarly, *Plasmodium* sp. MOLATE01 has been reported only at some places in the USA infecting brown-headed cowbirds (*Molothrus ater*) and house finches [53, 54]. We found this lineage infecting a new host, the canyon towhee. *Haemoproteus* sp. SISKIN1 is the most widespread lineage we found. It has been recorded in Lithuania, New York, California, Alaska, Russia and the Czech Republic, in a variety of hosts including *Carduelis spinus*, *Loxia curvirostra*, *Haemorhous mexicanus*, *Passer domesticus*, *Carpodacus erythrinus* and *Carduelis pinus* [54, 55]. Since all these lineages are widely distributed, future investigations should aim at determining the role that both resident and migrant species play in the infectious process in this region.

The similarity analysis showed that two of the sequences obtained had a difference of eight nucleotides compared to the sequence of the closest lineage (*Parahaemoproteus* ICTLEU01), and more differences regarding *Plasmodium* and *Haemoproteus* lineages. According to Bensch et al. lineage delimitation criteria [26, 29], this result suggests the presence of a lineage that has not been previously reported. Therefore, our results contribute to the knowledge of intraspecific diversity of these parasites.

## Conclusion

Haemosporidian parasite diversity in the highland plateau of San Luis Potosi includes at least four lineages from two genera, plus two sequences from a lineage that has not been reported previously. Parasitemia varies tremendously between individual species. While some suffer from high parasitemia, for unknown causes, others seem not to be parasitized. According to our results, habitat degradation by the establishment of small villages, and the associated tree extraction and overgrazing in the immediacies of these villages seems to increase parasitemia. Consequently, management practices for the mitigation of these negative effects near the villages such as maintenance of native trees and shrubs within villages, reforestation practices with native trees and shrubs in highly degraded shrublands, and the control of browse by domestic animal species could probably help in controlling parasitemia.

## Additional file

Additional file 1: GenBank accession numbers. Sequences 1 to 10 correspond to reference sequences in the NJ cladogram, 11 to 27 correspond to sequences obtained from samples of the canyon towhee (*M. fusca*) and 28 to 72 are sequences obtained from samples of the house finch (*H. mexicanus*). Sequences in bold are shown in the NJ cladogram. (CSV 4 kb)
